# Generation of a non-small cell lung cancer transcriptome microarray

**DOI:** 10.1186/1755-8794-1-20

**Published:** 2008-05-30

**Authors:** Austin Tanney, Gavin R Oliver, Vadim Farztdinov, Richard D Kennedy, Jude M Mulligan, Ciaran E Fulton, Susan M Farragher, John K Field, Patrick G Johnston, D Paul Harkin, Vitali Proutski, Karl A Mulligan

**Affiliations:** 1 Almac Diagnostics Ltd, 19 Seagoe Industrial Estate, Craigavon, BT63 5QD, UK; 2 Roy Castle Lung Cancer Research Programme, The University of Liverpool Cancer Research Centre, 200 London Road, Liverpool, L3 9TA, UK; 3 Centre for Cancer Research and Cell Biology, Queen's University of Belfast, 97 Lisburn Road, Belfast, BT9 7BL, UK

## Abstract

**Background:**

Non-small cell lung cancer (NSCLC) is the leading cause of cancer mortality worldwide. At present no reliable biomarkers are available to guide the management of this condition. Microarray technology may allow appropriate biomarkers to be identified but present platforms are lacking disease focus and are thus likely to miss potentially vital information contained in patient tissue samples.

**Methods:**

A combination of large-scale in-house sequencing, gene expression profiling and public sequence and gene expression data mining were used to characterise the transcriptome of NSCLC and the data used to generate a disease-focused microarray – the Lung Cancer DSA research tool.

**Results:**

Built on the Affymetrix GeneChip platform, the Lung Cancer DSA research tool allows for interrogation of ~60,000 transcripts relevant to Lung Cancer, tens of thousands of which are unavailable on leading commercial microarrays.

**Conclusion:**

We have developed the first high-density disease specific transcriptome microarray. We present the array design process and the results of experiments carried out to demonstrate the array's utility. This approach serves as a template for the development of other disease transcriptome microarrays, including non-neoplastic diseases.

## Background

Lung cancer is the single largest cause of cancer mortality worldwide [1]. The majority of lung cancer cases (70–85%) are non-small cell lung carcinoma (NSCLC), characterised by low treatment response rates and poor overall prognosis with 5-year survival of 15% and survival rate rarely exceeding one year [2]. Accurate staging of the disease is critical for the identification of the best treatment modalities and means of managing the disease [3]. However, the standard Tumour, Nodes, Metastasis (TNM) system used by pathologists in this respect is imprecise and limited information on prognosis [4] and the potential benefit from adjuvant or neo-adjuvant chemotherapy.

In recent years there has been an increasing focus on understanding the molecular basis of NSCLC [5]. A number of genes including *p53*, *RRM1 *and *ERCC1 *have been identified as being important in the development and progression of NSCLC [6,7]. A cluster of tumour suppressor genes at region *3p21.3 *have been indicated as inhibiting growth of lung cancer cells, and deletions in this region are commonly seen in lung and other cancers [8,9]. Other studies have examined the utility of molecular markers like *EGFR *and *HER2 *status in predicting patient survival and response to chemotherapy [1,10-12] and suggested that NSCLC is a prime candidate for targeted/individualised therapy [13-16].

Recent developments in the field of molecular genetics have highlighted the complexity of the transcriptome, questioning many of the fundamental assumptions about genes and their regulation [17-20]. This complexity is primarily due to processes such as the alternative splicing and polyadenylation of coding transcripts and the presence of non-coding and antisense transcripts, with non-coding transcripts thought to make up a substantial proportion of the human transcriptome [21,22]. The mammalian transcriptome has been estimated to consist of as many as 10^7 ^RNAs [23] and it has been suggested that the transcript rather than the gene should be regarded as the operational unit of the genome [24]. This is of particular relevance to lung tissue, which has been shown to have a high degree of transcriptome complexity relative to other tissues [25,26].

Commonly used gene expression profiling tools are sub-optimal for the study of lung tissue as they focus on well-characterized genes, resulting in the omission of a significant amount of information potentially of biological importance within the lung transcriptome. An expression array capable of detecting the entire human transcriptome would yield substantial benefits; however, this is unachievable with the current capacity limitations of microarray technology.

Therefore, focusing on the generation of gene expression arrays comprehensively representing the transcriptomes of individual tissues and diseases is a more feasible approach with current technology platforms. Using this approach we have characterized the transcriptome of Non-Small Cell Lung Carcinoma (NSCLC) and normal lung tissue and used this information to develop a disease focused microarray – the Lung Cancer DSA research tool. We believe that this platform enables comprehensive transcriptome level expression profiling of lung tissue samples.

## Methods

### Array generation

#### cDNA library generation

The NSCLC tumor libraries were generated from a set of frozen tissue samples including ~60% adenocarcinomas and ~40% squamous cell carcinomas and representing all TNM stages, derived from 65 male and female patients. The normal library was generated from a set of frozen normal lung tissues obtained from 19 male and female donors representing a mix of ethnicities and an age range of 32–65 years. All samples were collected following consultation with the University of Liverpool Committee on Research Ethics and with the written consent of all participating patients.

Samples of frozen lung tissue were homogenized in RNA STAT-60 (Tel-Test), and RNA was extracted according to the manufacturer's instructions. Equal amounts of good quality total RNAs were pooled and mRNA was isolated using the *μ*MACS mRNA isolation kit (Miltenyi Biotec) as described by the manufacturer. Non-radiolabeled lung cDNA libraries were constructed from 3 *μ*g of mRNA using the CloneMiner™ cDNA library construction kit (Invitrogen) according to manufacturer's instructions. The titer and average insert size in each cDNA library was determined according to the manufacturer's instructions and plasmid preparations of individual clones were carried out using a modified Montáge^® ^alkaline lysis method (Millipore) that incorporates MultiScreen^® ^Plasmid384 Miniprep clearing plates for centrifugal lysate clearing.

#### Sequencing of lung cDNA libraries

Cycle sequencing reactions were performed in 10 *μ*l volumes using a 1/16 dilution of Big Dye Terminator v3.1 ready reaction mix in Big Dye sequencing buffer (Applied Biosystems Inc.), 5 *μ*M M13 primer and 100 ng template DNA. Cycle sequencing was performed for 40 cycles at 95°C for 10 sec; 50°C for 5 sec and 60°C for 2 min 30 sec. Excess dye terminators were removed using CleanSEQ (Agencourt Biosciences). Sequence plates were analyzed on Applied Biosystems 3730/3730 × l DNA Analyzers using Applied Biosystems Sequence Analysis software.

#### Retrieval of public lung sequences

Human lung EST libraries were retrieved using the cDNA Library Finder at the National Cancer Institute's Cancer Genome Anatomy Project (CGAP) [27] website [28] (All cDNA libraries were retrieved from the CGAP website. Libraries annotated as originating from pooled tissue were excluded.

#### Inclusion of gene expression data

A total of 870 annotated microarray profiles generated on HG-U133 Plus 2 arrays were retrieved from the International Genomics Consortium (exp*O*) website [29] and processed using custom Perl scripts. 60 profiles originating from lung cancer samples were identified and probesets called present by the Affymetrix MAS5 algorithm in ≥ 10% of those were selected. In-house expression profiling of 5 normal and 5 tumor lung frozen samples was performed on HG-U133 Plus 2 arrays using standard Affymetrix protocols. Profiles were screened for MAS5 present calls and probesets called present at least once were combined with the probesets selected from exp*O *data. Full-length sequences corresponding to the selected Affymetrix probeset identifiers were downloaded from the Affymetrix website [30] and separated into polyadenylated and non-polyadenylated sequence groupings using Paracel Filtering Package.

#### Literature mining

A non-redundant list of genes previously associated with lung cancer was composed by means of Pubmed searches, subsequent literature review and by use of GeneGO's Metacore curated knowledgebase [31]. IDs of literature-derived genes were used to retrieve corresponding full-length mRNA sequences from the RefSeq [32,33], EMBL nucleotide sequence [34,35] and Ensembl [36] databases. The complete antisense complements to retrieved sequences were generated computationally.

#### Processing of public and in-house sequences

The 5' ESTs were filtered using Paracel Filtering Package (Paracel Inc.). Mitochondrial, bacterial and ribosomal contaminants as well as vector sequences, polyA tails, ambiguous end-regions and ESTs shorter than 100-bases were removed. Masking was performed for low-complexity regions (LCRs) and repeat sequences. The 3' ESTs were converted in to sense orientation using SeqUtil (Paracel Inc.) and filtered similarly to 5' ESTs, except 3' ESTs not containing polyA tails (8 or more consecutive adenine bases) were removed and masking of repeat sequences and LCRs was not performed in order to facilitate subsequent identification of alternative polyadenylation.

Paracel Transcript Assembler (PTA) (Paracel Inc.), a modified version of the CAP3 program [37] was used with default settings to assemble filtered 5' ESTs. The filtered 3' ESTs were also assembled using PTA but with the sequence-clipping option disabled and annotation of LCRs and repeats enabled in order to prevent spurious clustering and modification of the input sequences and to facilitate subsequent identification of alternative polyadenylation.

#### Detection of internal priming in 3' contigs and singlets

3' derived contigs and singlets were BLASTed against the RefSeq and EMBL databases. All BLAST analyses reported here were performed using Paracel Blast with e-value < 0.1. BLAST results and sequence files were processed using custom Perl scripts. Only same orientation alignments with sequence identity ≥ 95% over at least 100 bp (80 bp for singlets) and with <5 mismatches at their 3' extremity were considered. Alignment end-points (i.e. the last target base position matching the query sequence) were determined. Multiple alignment end-points occurring within a 300 bp window were clustered to produce a single end-point. The regions of target database sequences immediately downstream of the alignment end-points were analyzed for the presence of potential internal priming sites i.e. 8 or more adenine bases in a 10-base window.

#### Detection of alternative polyadenylation in contigs derived from 3' sequencing

All 3' ESTs used in the assembly were BLASTed against the contigs resulting from the assembly. Only same orientation alignments with sequence identity ≥ 95% and <26 mismatches at the 5' extremity and <5 mismatches at the 3' extremity of the query sequence were considered. Alignment end-points were determined and multiple end-points within a 300 bp window were clustered to produce a single end-point. Alignments ending <300 bp from a contig's 3' end were disregarded. The contig regions immediately downstream of the end-points were analyzed for the presence of potential internal priming sites and disregarded if internal priming appeared likely. Otherwise contigs were cleaved to produce alternatively polyadenylated forms.

#### 3'extension of sequences

Completeness of 3' coverage was uncertain for contigs derived from the 5' EST assembly and for non-polyadenylated sequences derived from expression data. In order to ensure proximity to a polyA tail and compatibility with 3' biased RNA amplification and labelling protocols an attempt was made to extend these sequences by aligning them to sequences from public databases. Where contig sequences could be extended in the same orientation, the corresponding public database sequences were chosen over the original sequences for array inclusion. When reverse orientation alignments were observed, both the original contigs and public database sequences were included. Public database sequences extending the 3' end of the expression data-derived sequences were included in the array design process along with the original expression data-derived sequences, since the latter were detected experimentally and likely represented alternatively polyadenylated or splice forms. Sequences not producing significant alignments to any public database sequences were included in their original form.

Contigs derived from the 5' EST assembly and non-polyadenylated sequences derived from expression data were BLASTed against sequences from RefSeq, EMBL and the 3' portion of UTRdb [38,39] databases. Alignments with ≥ 90% identity over ≥ 50% of the query sequence were selected and processed using custom Perl scripts in order to identify 3' extensions.

#### Sequence pruning and array design

All sequences were grouped and ranked according to their origin, quality and certainty of 3' end completeness. The 300-base long 3'-terminal regions of sequences from lower priority groupings were iteratively BLASTed against corresponding regions of sequences from all higher priority groupings and those producing alignments with ≥ 90% identity over ≥ 115 bp were removed. [See Additional file 1].

The design of standard 11 × 25-mer probe probesets was carried out by Affymetrix within the 300-base region at the 3' end of selected sequences. The subsection of this region to which probes were actually designed is referred to henceforth as a *target sequence*. Sequences were removed from the design if creation of at least 8 probes was not possible. All standard HG-U133 Plus 2 normalization and housekeeping control probesets were included in the design and custom versions designed within the last 300 bases were also requested.

### Sequence content analysis of the Lung Cancer DSA

#### Sequence annotation

Lung Cancer DSA research tool sequence content was annotated by blasting all target sequences against a series of public databases. The databases utilized (in order of priority) were RefSeq, human EMBL, human DDBJ and Unigene [40]. Target sequences were blasted and the highest prioritized database sequence they aligned to with ≥ 90% identity over ≥ 50% of their length was used for annotation. Annotation was performed by retrieval of a target sequence's corresponding public database accession number and publicly available annotation information associated with it. Where sequences did not produce a satisfactory alignment to any of these databases, alignments to the human genome were performed.

#### Derivation of unique content list

HG-U133 Plus 2 full sequences and probes were downloaded from the Affymetrix website [30]. Agilent and Illumina probes were downloaded from their respective manufacturers' websites [41,42]. Agilent and Illumina full sequences were unavailable from the manufacturers who recommended downloading representative sequences from public databases based on array annotation. Array annotation was retrieved from the manufacturers' websites and public sequence accession numbers representing the probesets on the Illumina and Agilent arrays were used to retrieve sequences where possible. All Illumina sequences were obtained using the Batch Entrez [43] nucleotide retrieval function at the NCBI website. A majority of Agilent sequences were retrieved in an identical fashion. A further subset of Agilent sequences was extracted using predicted transcript files (release 46) retrieved from Ensembl [44] and the DFCI's human gene index release 17.0 [45].

The Lung Cancer DSA probesets were blasted against the full sequences used in design of the generic arrays. Where 6 or more probes from a probeset (usually 11 probes) aligned to the same sequence with 100% identity over their entire length, the probeset was considered 'common' to a generic array. Full sequences representing probesets not considered common at this stage were extracted and generic array probes blasted against them. For the HG-U133 Plus 2 array, 6 or more probes from a probeset (usually 11 probes) aligned to the same sequence with 100% identity over their entire length was considered a positive result. Since the Agilent and Illumina platforms utilize single probes rather than probesets, a single probe alignment of 100% identity across its entire length was considered a positive result. Where one or more of the 3 generic arrays produced a positive result, the Lung Cancer DSA probeset representing the full sequence was again considered 'common'. Thus, the 'unique' grouping represents probesets that do not bear significant similarity to a generic array's full sequence, nor do they show significant similarity between their own full sequences and the generic arrays' probes.

#### Gene Ontology analysis

A selection of major biological process categories relevant to cancer studies were selected and all related biological process GO terms retrieved using the AmiGO browser and search engine on the Gene Ontology website [46]. The 9 major categories selected were: angiogenesis, apoptosis, proliferation, cell-cycle control, developmental processes, DNA repair, cell signaling, cell migration and immunology/inflammation. 535 biological process GO terms were retrieved and associated with the category they belonged to. A custom Perl script was used to search the unique Lung Cancer DSA probesets' annotations for GO terms and associate probesets with *each *category for which their annotation contained a related term. Probesets whose annotation did not contain GO terms or contained GO terms not associated with the major categories were classified in an "unknown" or "other" grouping respectively. Lists of those probesets detected, differentially expressed, or annotating in reverse orientation to RefSeq sequences were created and used by the script to further subdivide the 9 categories.

### Technical assessment experiment

#### Tissue collection and RNA isolation

Frozen pairs of lung squamous cell carcinoma and adjacent normal lung tissue originating from a single donor were obtained from Asterand (Detroit, MI). All sample pairs were processed immediately and under identical conditions. All Asterand samples are collected following written patient consent and ethical review board approval [47].

Total RNA was isolated from frozen samples using Stat-60 (Tel Test, Friendswood, TX). Following RNA isolation, the frozen samples were subjected to DNase treatment using the RNase-free DNase set (Qiagen) and then purified and concentrated using the RNeasy MinElute Cleanup Kit (Qiagen).

#### Target preparation, hybridization and Affymetrix GeneChip analysis

Target preparation was performed using the WT-Ovation™ RNA Amplification System (NuGEN Technologies, San Carlos, CA). To ensure sufficient cDNA yield, ten replicate amplifications were performed from each starting RNA sample using 10 ng and 50 ng of total RNA from the frozen samples. The amplified cDNA was fragmented and labeled using the FL-Ovation™ cDNA Biotin Module V2. Randomly selected pairs of fragmented cDNA samples were pooled and 5 *μ*g of cDNA hybridized to Lung Cancer DSA arrays. Arrays were then washed and stained using Affymetrix fluidics script EukGE-WS2-v4 and scanned using the Affymetrix GeneChip Scanner 3000 for data acquisition. All kits, reagents and equipment were used according to manufacturer's instructions.

The yield of total RNA and amplified cDNA was assessed using the Eppendorf Biophotometer. The quality of the total RNA, cDNA and fragmented targets was determined using the Agilent 2100 Bioanalyser according to manufacturer's instructions.

#### Gene expression analysis

Quality of samples and data was assessed on the basis of parameters extracted from GCOS report (RPT) files and detection of array outliers performed with the dChip software [48] Version 2007.

Gene expression analysis was carried out with dChip software and Matlab^® ^(Version 2007a) with Bioinformatics and Statistics Toolboxes. Data pre-processing was carried out with dChip Invariant Set Normalization and PM-only Model Based Expression summarization. The MAS5.0 algorithm with the default significance threshold (*α *= 0.05) was used to define present calls for probesets.

Coefficient of variance and correlation coefficient were calculated only for probesets consistently called present in all tumor and all normal replicates. Coefficient of variation for replicates in normal and tumor groups was calculated as a median value of the ratios of standard deviation of intensity to the median intensity for all qualifying probesets across replicates, and multiplied by 100 to produce the Coefficient of Variance. The correlation coefficient was calculated as the average value of the Spearman correlation coefficients for all pairs of replicates within normal and tumor groups. Autocorrelations were excluded.

Selection of differentially expressed probesets (DEPS) between the replicates of tumor and normal samples was performed using the following criteria:

(1) Fold change (*FC*) filter: |log_2_(*FC*)| > log_2_(1 + 3*μCV) = log_2_(1.2), where *μCV *is the median of the coefficients of variation of expression intensity (*CV*_*i*_) for all probesets on the array. *CV*_*i *_for each probeset was calculated as a ratio of the pooled standard deviation of probeset intensity to the median probeset intensity: $CV_{i}=\frac{1}{\mu_{i}}\sqrt{\frac{\left(n_{1}-1\right)\sigma_{i1}+\left(n_{2}-1\right)\sigma_{i2}}{n_{1}+n_{2}-2}}$, where *μ*_*i *_is the median probeset intensity for all replicates in both normal and tumor groups; *σ*_*i*1 _and *σ*_*i*2 _are the standard deviations of probeset intensity and *n*_1 _and *n*_2 _are the numbers of replicates in normal and tumor groups, respectively.

(2) Low expression difference filter: |*E *- *B*| > [AvBg + 3* *σ*(Bg)] = 38, where *E *and *B *are average expression intensities in tumor and normal groups, respectively; AvBg is the average background value for all profiles in tumor and normal groups; and *σ*(Bg) is standard deviation of background values for all profiles in tumor and normal groups.

(3) Present call filter: present in all replicates of an over-expressed group (tumor or normal).

(4) Student's t-test filter: p < 0.001.

Perl and Matlab scripts used for data processing and analysis are available from the authors upon request.

## Results and Discussion

### Generation of the Lung Cancer DSA

**Figure 1:**
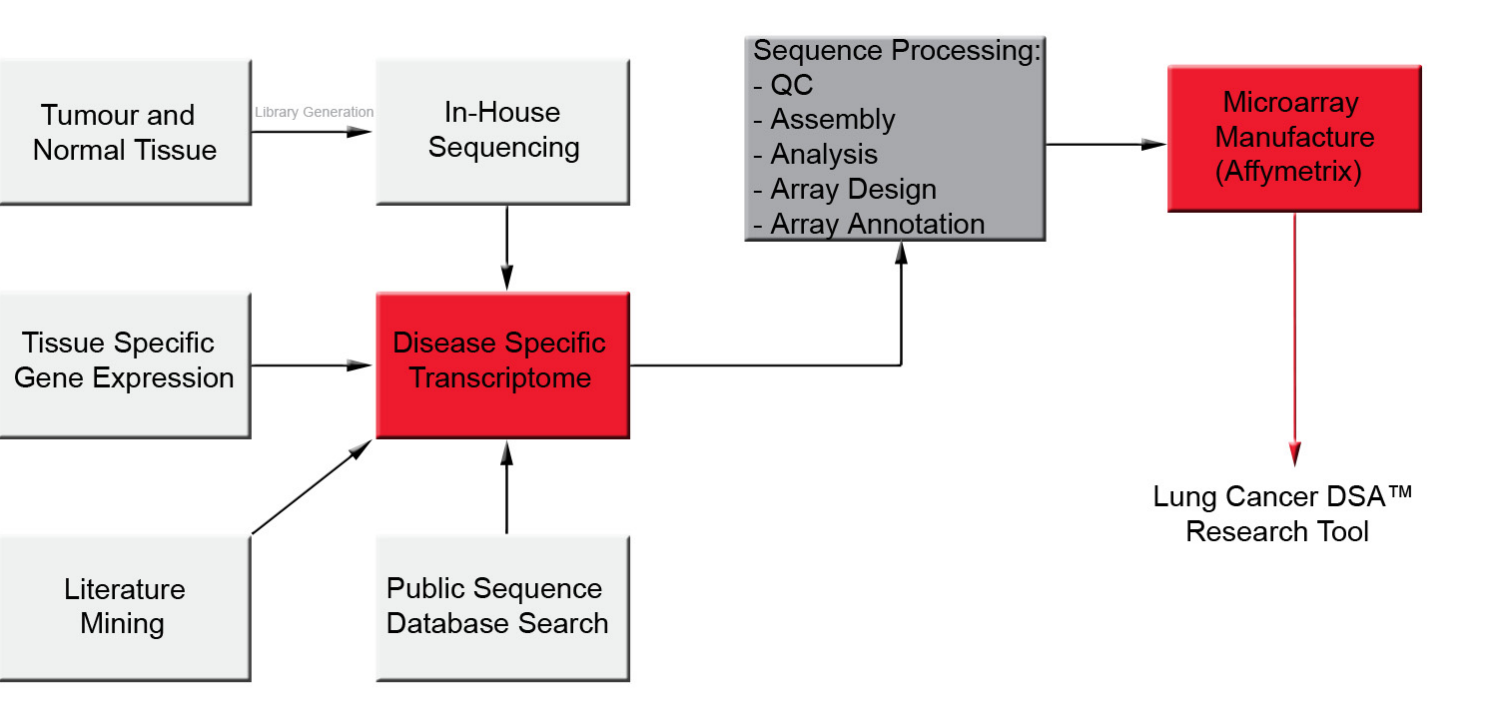
Schematic representation of the generation of the Lung Cancer DSA research tool showing the parallel approaches (in-house sequencing, public sequence database mining and gene expression profiling) used to characterize the NSCLC transcriptome, from which the Lung Cancer DSA was designed.

The transcriptome of NSCLC and normal lung tissue was characterized using three sources of information (Figure 1)

#### (i) In-house and publicly available sequence data

Non-normalized cDNA libraries were generated and sequenced separately for NSCLC tumor and normal lung tissue. 403,494 expressed sequence tags (ESTs) were generated in-house from the tumor libraries and supplemented by 336,280 human lung ESTs from 291 publicly available libraries in order to optimally represent the diversity of the NSCLC transcriptome. These 3' and 5' derived sequences were included in the array design process along with 166,411 3' ESTs generated in-house from the normal lung library. The 3' and 5' derived sequences were filtered and assembled separately producing a total of 41,497 contigs and 89,695 singlet sequences for the array design process.

#### (ii)Gene expression data derived from in-house experiments and public databases

Data from 70 publicly available and in-house generated lung normal and cancer microarray profiles were analyzed in order to identify transcripts reliably detected as expressed in lung tissue. As a result, 17,128 polyadenylated and 21,687 non-polyadenylated publicly available sequences corresponding to the detected probesets were selected for the array design process.

#### (iii) Literature mining

In addition to the above empirical approaches we performed literature mining, which resulted in the inclusion of 1,445 full-length mRNA sequences previously implicated in lung cancer. This grouping contained both known and putative transcripts. Analysis revealed that 96% of these had already been identified by the empirical means described, demonstrating the comprehensive nature of the approach. Due to their potential importance, the literature-derived sequences were retained as a distinct grouping in order that they be excluded from pruning. We also included their computationally derived antisense complements.

The sequence data obtained from these approaches was assembled and analyzed for internal priming, alternative polyadenylation and potential 3' termini extension of 5' sequences

### Identification of alternative polyadenylation

The advantage of 3' sequencing is that it allows the capture of genuine 3' sequence termini and the detection of alternative polyadenylation. This important information is usually lost as a result of applying conventional sequence assembly methods. In order to prevent this loss, contigs derived from 3' in-house sequences were examined, using a custom computational pipeline, for potential instances of alternative polyadenylation. 732 such instances were identified and alternatively polyadenylated forms of such contigs were also included in the array design process. The combined approach of 3' sequencing and computational identification of alternative polyadenylation ensured that the final array represented all detectable 3' splice/polyadenylation variants identified by our methods. This group of sequences potentially contains tissue or disease specific variants vital to focused NSCLC studies and not represented on generic microarrays.

### Sequence pruning and array design

All selected sequences were grouped and ranked according to their origin, quality and certainty of 3' end completeness and pruned against one another to remove redundancy [See Additional file 2]. The literature-derived lung cancer related sequences were given the highest priority and were not pruned. The resulting non-redundant set of sequences was considered to represent the NSCLC transcriptome and 3' target regions of these sequences were submitted to Affymetrix for probe design and array manufacture.

### Sequence content assessment of the Lung Cancer DSA

**Figure 2:**
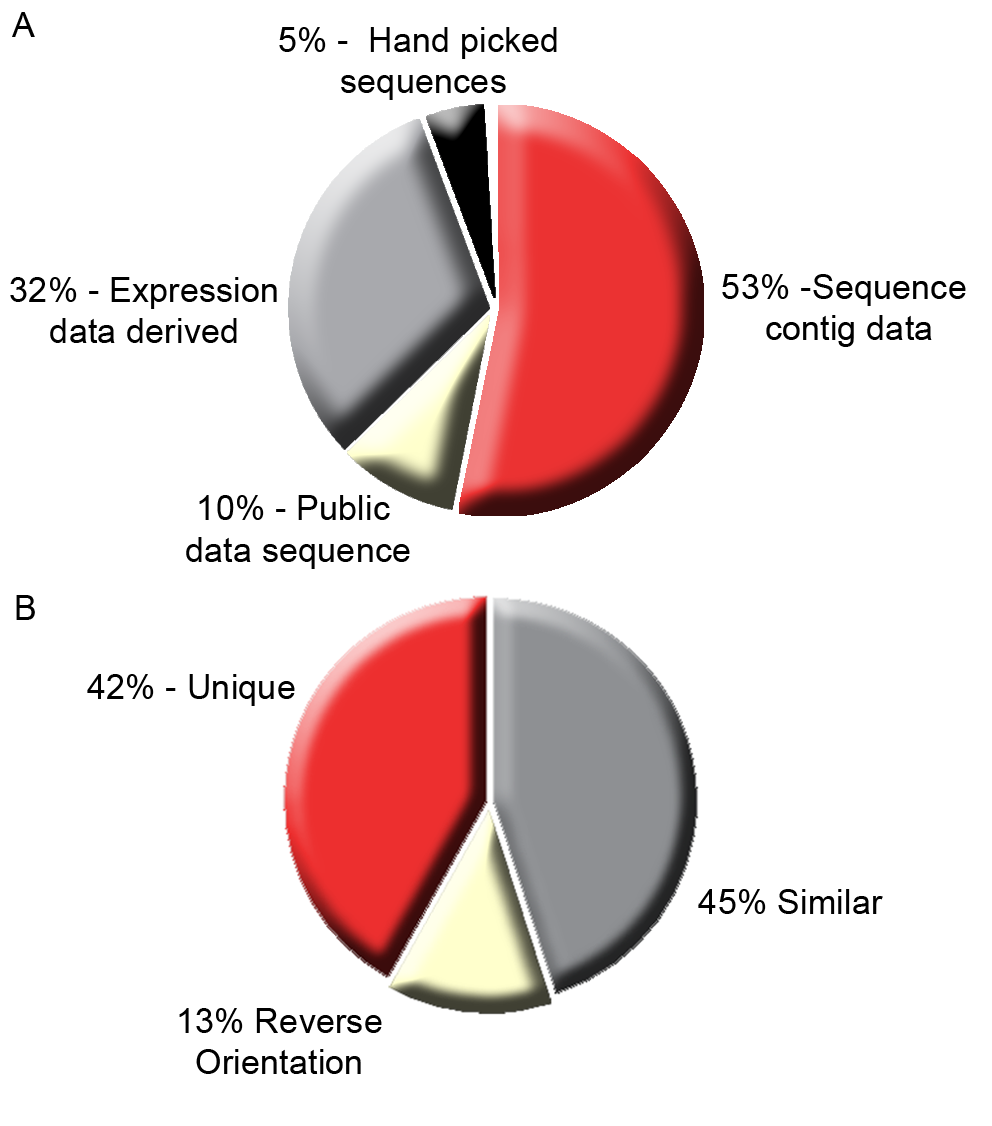
**A**) Origin of the content of the completed Lung Cancer DSA, showing that the majority of the array content (53%) was derived from sequencing data, followed by expression profiling derived data (32%). **B) **Comparison of the Lung Cancer DSA sequence content with the RefSeq mRNA database.

The resulting Lung Cancer DSA research tool contains 59,927 probesets representing transcripts expressed in NSCLC and normal lung tissue, and a further 489 normalization, hybridization and housekeeping control probesets, including all 162 standard Affymetrix HG-U133 Plus 2 GeneChip (HG-U133 Plus 2) controls [See Additional file 2]. The majority of the array content (53%) was derived from cDNA sequencing (Figure 2A). This was followed by expression data-derived sequences (32%) and public database sequences identified while extending 3' termini of 5' EST assembly and expression data derived sequences (10%). The remaining 5% of the array content consists of literature-derived sequences previously implicated in lung cancer and their antisense complements.

Analysis of the array content demonstrated that 42% of transcripts on the Lung Cancer DSA research tool have no significant homology with sequences from NCBI's Reference Sequence Database, RefSeq in either orientation and 13% represent sequences transcribed in antisense orientation to annotated RefSeq transcripts (Figure 2B). Further analysis identified a total of 6,206 transcripts, represented on the Lung Cancer DSA research tool in both the sense and corresponding antisense orientation.

Comparison of the content of the Lung Cancer DSA research tool with the 3 leading commercial human arrays demonstrated that there were 18,635 transcripts, 27,777 transcripts and 31,211 transcripts not detectable by the Affymetrix HG U-133 Plus2 array, Agilent Whole Human Genome Array and the Illumina Human 6 array respectively (Table 1). In addition, a core set of 15,541 Lung Cancer DSA research tool unique transcripts were not detectable by any of the 3 commercial arrays, thus demonstrating the increased coverage of the lung transcriptome by the Lung Cancer DSA research tools.

**Table 1:** Comparison of the Lung Cancer DSA content with the Affymetrix Plus2, Agilent Whole Human Genome and Illumina Human 6 arrays.

**Commercial gene expression microarray**	**Lung Cancer DSA research tool unique transcripts**
Affymetrix HG-U133 Plus2 array	18635
Agilent Whole Human Genome array	27777
Illumina Human 6 array	31211
Comparison with content of 3 arrays combined	15541

Examination of these 15,541 transcripts revealed that 4% (647) had significant homology with RefSeq transcripts, 40% (6,157) had significant homology with RefSeq in antisense orientation and 21% (3,302) and 16% (2,420) had significant homology with EMBL in sense and antisense orientations respectively (Table 2). For the remaining transcripts 16% (2,514) had significant homology with Unigene and 2% (353) with the human genome, while 148 demonstrated no homology with any of the databases. The large number of antisense sequences included within this grouping is notable. Antisense transcription is a subject which is now receiving increased attention in scientific literature and these transcripts are underrepresented on current generic microarrays. Antisense transcription has been associated with many important regulatory functions and its consideration when designing the Lung Cancer DSA research tool has ensured inclusion of probesets potentially vital in applications such as classifier generation.

**Table 2:** Experimental detection of the Lung Cancer DSA unique content in matched normal tumor lung tissue.

**Annotation database**	**# Unique transcripts**	**# Detected unique transcripts**	**# Differentially expressed unique transcripts**
RefSeq	647	155	43
Antisense to RefSeq	6157	3377	1301
EMBL	3302	982	274
Antisense to EMBL	2420	852	256
Unigene	2514	1021	306
Genome	353	9	4
Unannotated	148	2	0

### Technical assessment experiment

To assess the biological relevance of the content of the Lung Cancer DSA research tool, five technical replicates of two RNAs extracted from a single patient matched normal and NSCLC frozen tissue were profiled on the arrays. The results demonstrated that in total 35,625 transcripts were consistently detected by the Lung Cancer DSA research tool as being expressed in either the normal or tumor tissue. These included 6398 (41%) of the Lung Cancer DSA research tool unique transcripts with 155 RefSeq, 982 human EMBL and 1,021 Unigene transcripts being detected in the sense orientation (Table 2). In addition we observed significant detection of antisense transcripts, with 3,437 (55%) of the RefSeq sense-antisense (SA) transcript pairs detected as being expressed in either the normal or tumor tissue.

Comparing the transcript expression levels between the normal and tumor lung tissue identified 2,148 of the unique transcripts as being differentially expressed and thus potentially important to the underlying biology of this disease (Table 2). These included RefSeq sense transcripts to predicted proteins with associated functions in processes such as apoptosis, cell cycle control, cell proliferation and DNA damage repair [See Additional file 3]. In addition there was extensive differential expression of sense transcripts with homology to the coding DNA sequence (cds) of known genes and antisense transcripts (Table 2).

To further investigate the relevance of the detected and differentially expressed unique content to the biology of NSCLC, the annotation associated with these transcripts was assessed by Gene Ontology mining for implicated roles in processes linked to cancer. This clearly demonstrated association of these transcripts with the main cellular processes linked to cancer, including proliferation, apoptosis and DNA damage repair (Table 3). An example of one such group, DNA repair, is given in Table 4 where 21 antisense transcripts differentially expressed between the normal and tumor samples were identified.

**Table 3:** Gene Ontology mining of the experimentally detected unique Lung DSA transcripts associated annotation.

**GO process**	**Unique detected transcripts**	**Unique detected RefSeq antisense transcripts**	**Unique differentially expressed transcripts**	**Unique differentially expressed RefSeq antisense transcripts**
Angiogenesis	26	18	13	9
Apoptosis	311	186	112	77
DNA repair	119	65	32	21
Cell migration	71	34	27	17
Proliferation	287	186	117	85
Immunology/Inflammation	196	118	83	55
Developmental genes	12	8	10	7
Cell cycle control	384	239	139	111
Cell Signaling Pathway	55	40	33	25
Other	3851	2270	1382	910
Unknown	2461	820	770	293

**Table 4:** RefSeq antisense transcripts differentially expressed between the normal and tumor lung tissue, from the unique Lung Cancer DSA research tool content.

**Target accession number**	**Probe ID**	**Database**	**Orientation**	**Gene symbol**	**Fold change**	**P value**
NM_000546	LCHPRC.1183_s_at	RefSeq	Antisense	TP53	2.12	0.000147
NM_000059	LCHPRC.7_at	RefSeq	Antisense	BRCA2	2.73	0.000056
NM_130398	LCMXR.3025C1_at	RefSeq	Antisense	EXO1	4.22	0.000566
NM_002431	LCHPRC.358_at	RefSeq	Antisense	MNAT1	1.89	0.000006
NM_078468	LC3P.8284C2_at	RefSeq	Antisense	BCCIP	1.80	0.00001
NM_002129	LCSS.2843_at	RefSeq	Antisense	HMGB2	1.66	0.000061
NM_007192	LCMXR.12622C1_at	RefSeq	Antisense	SUPT16H	1.82	0.000001
NM_001239	LCHPRC.1335_at	RefSeq	Antisense	CCNH	-1.95	0.000007
NM_004219	LCMXR.7995C1_at	RefSeq	Antisense	PTTG1	3.7	0.000000
NM_001274	LCHPRC.250_at	RefSeq	Antisense	CHEK1	6.63	0.000013
NM_001806	LCMXR.17153C1_at	RefSeq	Antisense	CEBPG	2.82	0.000123
NM_000057	LCHPRC.1353_at	RefSeq	Antisense	BLM	1.68	0.000804
NM_021117	LCMXR.1610C3_at	RefSeq	Antisense	CRY2	-5.89	0.000007
NM_001067	LCHPRC.222_at	RefSeq	Antisense	TOP2A	10.81	0.000002
NM_000123	LCMXR.7050C1_at	RefSeq	Antisense	ERCC5	-1.99	0.000014
NM_014502	LCMXR.187C1_at	RefSeq	Antisense	PRPF19	1.82	0.000085
NM_021117	LCMXR.1610C1_s_at	RefSeq	Antisense	CRY2	-3.4	0.000341
NM_001274	LCMXR.7773C1_at	RefSeq	Antisense	CHEK1	7.07	0.000022
NM_002945	LCMXR.7467C1_at	RefSeq	Antisense	RPA1	1.7	0.00008
NM_007027	LCMXR.5891C1_at	RefSeq	Antisense	TOPBP1	4.74	0.000004
NM_152221	LCMXR.392C1_at	RefSeq	Antisense	CSNK1E	1.57	0.000041

## Conclusion

The recently published results of the ENCODE consortium's pilot project indicate that the majority of the human genome is transcribed and that a minority of transcriptional activity leads directly to protein production [19]. This and other evidence has highlighted the complexity of the transcriptome and the need for better tools to study it [23,49]. Since studying the entire human transcriptome is not possible using current technologies, we propose a practical alternative of developing a range of microarray research tools capable of interrogating transcriptomes of individual disease settings

Given the importance of NSCLC as a disease and the extent of genomics research in this area, we have endeavoured to characterise the transcriptome of NSCLC by means of in-house sequencing, mining of public sequence databases, gene expression profiling and literature mining. This information was used to design the Lung Cancer DSA representing ~60,000 transcripts empirically shown to be expressed in NSCLC and normal lung tissue

When the Lung Cancer DSA is compared with the three most commonly used generic gene expression microarrays, a significant proportion of the array is unique in relation to each array as shown in Table 1. 15,541 Lung Cancer DSA research tool transcripts are not detectable by the Affymetrix HG U-133 Plus2 array, the Agilent Whole Human Genome Array or the Illumina Human 6 array, clearly demonstrating that the transcriptome of NSCLC is better represented by the Lung Cancer DSA research tool. This enables researchers using the Lung Cancer DSA to interrogate over 15,000 additional transcripts, all relevant to NSCLC.

The technical assessment experiment shows that data generated by the Lung Cancer DSA is highly reproducible and exhibits extremely good correlation and low coefficient of variance, similar to those obtained from generic Affymetrix arrays in our own [See Additional file 4] and in previously published studies [50].

A large part of the Lung Cancer DSA content is not represented in the RefSeq database of well annotated mRNAs. Importantly, 46.3% of the non-RefSeq transcripts were reliably detected in the technical assessment experiment using samples taken from a single patient.

An even greater proportion (58%) of transcripts antisense to annotated RefSeq sequences were also consistently detected, further highlighting the magnitude of antisense transcription that is not fully annotated or understood. Yelin et al [51] and Chen et al [52] predicted that there were 2667 and 5880 sense-antisense (SA) pairs transcribed from the human genome respectively. Our results from the analysis of a single tissue type and disease setting would suggest that the total number of SA pairs in the human transcriptome may be considerably higher.

Interestingly, based on the hypothesis that antisense transcription is the norm rather than the exception, we included in the array design 1143 sequences artificially created as antisense to those derived by literature and pathway mining. Of these 1143 sequences, 412 were consistently detected in the technical assessment experiment with 198 of these being differentially expressed, lending weight to the growing body of evidence that antisense transcription is extremely widespread [51,53-55].

Gene ontology mining served to demonstrate the potential importance of the unique Lung Cancer DSA content. Major cancer related GO process categories were highly represented by uniquely detected or differentially expressed transcripts and significant proportions of these transcripts were shown to bear antisense homology to well characterised RefSeq sequences (Table 4). 21 antisense transcripts linked to DNA damage repair were differentially expressed between the normal and tumor samples. These included antisense transcripts homologous to BRCA2, CHEK1 and TP53. Interestingly, TP53 antisense mRNA has previously been identified in human cells [51]. Antisense sequences are believed to function in gene regulation by modulating sense mRNA transcription, maturation, transport, stability and translation. The extensive detection of differentially expressed antisense transcripts between the normal and tumor tissue supports a role for these sequences in NSCLC pathogenesis. Moreover, a conventional generic microarray would not have detected these potentially important transcripts.

The Lung Cancer DSA research tool presented in this communication represents a powerful and practical tool for both basic and translational research that better reflects the complexity of the NSCLC transcriptome than commonly used microarrays. We believe it to have applications in areas such as pathway analysis, biomarker and drug target discovery and multivariate prognostic and predictive classifier generation. Its disease-focused design and novel, relevant content could ultimately lead to a better understanding of the underlying biology of NSCLC.

In addition, this methodology serves as a template for the development of other disease transcriptome focused microarrays, including non-neoplastic diseases.

## Competing interests

The majority of the authors are employees of Almac Diagnostics – a commercial entity – and accordingly we declare potential financial competing interests.

## Authors' contributions

AT participated in the DSA research tool and study design, drafted the manuscript and was involved in coordinating the study. GRO participated in and performed design of the DSA research tool, performed the sequence content analysis and helped to draft the manuscript. VF performed gene expression analysis and participated in sample quality control and helped to draft the manuscript. RDK was involved in the study design and coordination. JMM designed and performed the microarray expression experimental work for the technical validation of the DSA research tool. CEF coordinated the sequencing of the lung EST libraries. SMF carried out RNA extractions and cDNA library generation

JKF was involved in the study design. PGJ was involved in the initial conceptualisation and design of the DSA research tool. DPH was involved in study design and the initial conceptualisation and design of the DSA research tool. VP was involved in the study design and coordination and helped to draft the manuscript. KAM was involved in the initial conceptualisation and design of the DSA research tool, participated in study design and coordination and helped to draft the manuscript.

## Pre-publication history

The pre-publication history for this paper can be accessed here:



## Supplementary Material

Additional file 1 Sequence content source breakdown for Lung Cancer DSA (table). Click here for file

Additional file 2 Lung Cancer DSA technical specifications (table) Click here for file

Additional file 3 RefSeq transcripts differentially expressed (table). Click here for file

Additional file 4 Reproducibility and reliability for technical study (table). Click here for file
