# Supplementary material for: Generation of a non-small cell lung cancer transcriptome microarray
Source: BMC Med Genomics. 2008 May 30;1:20. doi: 10.1186/1755-8794-1-20 (PMC2426710; doi:10.1186/1755-8794-1-20)
Supplement: Additional file 1 — Sequence content source breakdown for Lung Cancer DSA (table). [file 1755-8794-1-20-S1.doc]

# Supplementary Table 1

| **Sequence Groupings** | **Original number of sequences** | **Number of sequences pruned out** | **Number of retained sequences** |
| --- | --- | --- | --- |
| Literature derived sequences | 1445 | not pruned | 1445 |
| 3` contigs | 11064 | not pruned | 11064 |
| Alternatively polyadenylated versions of 3` contigs | 732 | not pruned | 732 |
| Public representative of 3` singlets | 7130 | 1808 | 5322 |
| Polyadenylated expression data-derived sequences | 17128 | 7315 | 9813 |
| 5` contigs | 8546 | 600 | 7946 |
| RefSeq sequences | 13220 | 8840 | 4380 |
| EMBL sequences | 1987 | 555 | 1432 |
| Non-polyadenylated expression data-derived sequences | 19104 | 7475 | 11629 |
| 3` singlets | 17705 | 8382 | 9323 |
| UTRdb sequences | 2507 | 1680 | 827 |
| Reverse complemented Literature derived Sequences | 1445 | 67 | 1378 |

Supplementary Table 1. Sources of sequence information for the design of the Lung Cancer DSA research tool.
