# Supplementary material for: Generation of a non-small cell lung cancer transcriptome microarray
Source: BMC Med Genomics. 2008 May 30;1:20. doi: 10.1186/1755-8794-1-20 (PMC2426710; doi:10.1186/1755-8794-1-20)
Supplement: Additional file 2 — Lung Cancer DSA technical specifications (table) [file 1755-8794-1-20-S2.doc]

# Supplementary Table 2

| Technical Specifications of the Lung Cancer DSA Research Tool | |
| --- | --- |
| Number of probesets | 60,416 |
| Feature Size | 11 |
| Probe Length | 25-mer |
| Probe Pairs/ Probeset | 11 |
| Affymetrix Normalization Controls | 100 |
| Affymetrix Hybridization/ Housekeeping Controls | 62 |
| Almac Diagnostics Controls | 327 |

Supplementary Table 2. Technical specifications of the Lung Cancer DSA research tool.
