# Supplementary material for: Generation of a non-small cell lung cancer transcriptome microarray
Source: BMC Med Genomics. 2008 May 30;1:20. doi: 10.1186/1755-8794-1-20 (PMC2426710; doi:10.1186/1755-8794-1-20)
Supplement: Additional file 3 — RefSeq transcripts differentially expressed (table). [file 1755-8794-1-20-S3.doc]

# Supplementary Table 3

| **Target Accession Number** | Probeset ID | **Orientation** | **Database** | **Gene Name** | **Fold change** | **P value** |
| --- | --- | --- | --- | --- | --- | --- |
| XM_001127703 | LCRS.7940_at | Sense | Refseq | PREDICTED: Homo sapiens hypothetical protein LOC730911 (LOC730911) | 4.57 | 0.000005 |
| XM_371638 | LC3SNGa.751a10_at | Sense | Refseq | PREDICTED: Homo sapiens similar to FAT tumor suppressor homolog 1 | -2.93 | 0.000055 |
| XR_017045 | LCMXR.6511C1_at | Sense | Refseq | PREDICTED: Homo sapiens hypothetical LOC645561 (LOC645561) mRNA. | 16.41 | 0.000002 |
| XR_015991 | LCSS.9029_s_at | Sense | Refseq | PREDICTED: Homo sapiens similar to 40S ribosomal protein S10 | 2.25 | 0.000001 |
| XM_376795 | LC3SNG.302a230_at | Sense | Refseq | PREDICTED: Homo sapiens FLJ45872 protein (FLJ45872) mRNA. | -9.67 | 0.000494 |
| XM_001127016 | LCSSRS2.8249_at | Sense | Refseq | PREDICTED: Homo sapiens hypothetical protein LOC728262 (LOC728262) | 3.02 | 0.000023 |
| XR_019075 | LCSSRS2.5231_at | Sense | Refseq | Homo sapiens similar to Adipophilin (Adipose differentiation-related protein) (ADRP) (LOC648660), mRNA | -1.87 | 0.000385 |
| XM_001126174 | LCMXR.12806C1_at | Sense | Refseq | PREDICTED: Homo sapiens hypothetical protein LOC727929 (LOC727929) | 2.38 | 0.00004 |
| XM_001127685 | LCSSRS2.3967_at | Sense | Refseq | PREDICTED: Homo sapiens similar to Baculoviral IAP | -1.97 | 0.000002 |
| XM_001126849 | LCRS.9436_at | Sense | Refseq | PREDICTED: Homo sapiens hypothetical protein LOC727900 (LOC727900) | 5.71 | 0.000006 |

Supplementary Table 3. RefSeq sense transcripts differentially expressed between the normal and tumor lung tissue, from the unique Lung Cancer DSA research tool content.
