# Supplementary material for: Generation of a non-small cell lung cancer transcriptome microarray
Source: BMC Med Genomics. 2008 May 30;1:20. doi: 10.1186/1755-8794-1-20 (PMC2426710; doi:10.1186/1755-8794-1-20)
Supplement: Additional file 4 — Reproducibility and reliability for technical study (table). [file 1755-8794-1-20-S4.doc]

# Supplementary Table 4

| Normal Tissue | | Tumour Tissue | |
| --- | --- | --- | --- |
| Coefficient of Variance | Correlation | Coefficient of Variance | Correlation |
| 5.2% | 98.59% | 5.3% | 98.70% |

# Supplementary Table 4. Table showing the coefficients of variation and the subgroup average correlation coefficients calculated for the Lung Cancer DSA in the technical assessment experiment.
